# Empowerment of Patients with Hypertension through BPM, IoT and Remote Sensing

**DOI:** 10.3390/s17102273

**Published:** 2017-10-04

**Authors:** Daniel Ruiz-Fernández, Diego Marcos-Jorquera, Virgilio Gilart-Iglesias, Víctor Vives-Boix, Javier Ramírez-Navarro

**Affiliations:** Department of Computer Technology, University of Alicante, 03690 Alicante, Spain; dmarcos@dtic.ua.es (D.M.-J.); vgilart@dtic.ua.es (V.G.-I.); vvives@dtic.ua.es (V.V.-B.); jramirez@dtic.ua.es (J.R.-N.)

**Keywords:** health devices, health sensors, Business Process Management, hypertension, IoT, remote sensing

## Abstract

Hypertension affects one in five adults worldwide. Healthcare processes require interdisciplinary cooperation and coordination between medical teams, clinical processes, and patients. The lack of patients’ empowerment and adherence to treatment makes necessary to integrate patients, data collecting devices and clinical processes. For this reason, in this paper we propose a model based on Business Process Management paradigm, together with a group of technologies, techniques and IT principles which increase the benefits of the paradigm. To achieve the proposed model, the clinical process of the hypertension is analyzed with the objective of detecting weaknesses and improving the process. Once the process is analyzed, an architecture that joins health devices and environmental sensors, together with an information system, has been developed. To test the architecture, a web system connected with health monitors and environment sensors, and with a mobile app have been implemented.

## 1. Introduction

High blood pressure (hypertension) is considered a risk for cardiovascular disease and has a high prevalence in the population [1]. Known as the “silent killer”, can present no symptoms and it contributes to damage the heart and blood vessels in organs such as brain and kidneys. According to the World Health Organization, one in five adults worldwide have high blood pressure and this condition is directly related with half of all deaths from stroke and heart disease [2]. In Spain, hypertension prevalence in people older than 65 years old has been increasing progressively in the last decades, from 53.2% in 1993 to 86.2% in 2006 [3].

High blood pressure is preventable and the treatment includes both drugs and recommendations for a healthier lifestyle: healthy diet, physical activity and stress management [2]. Despite the importance of following the treatment and these recommendations, many patients forget them mainly because they feel healthy and they don’t realize the benefits for treating high blood pressure (HBP) and consequently, for their health [4]. In [5] we can find several factors related to the compliance with treatments such as the difficulty to understand leaflets, low awareness about treatment and complications of hypertension, doctor-patient interaction not encouraging, etc. Apart from the health problems for the patients derived from a low compliance there are other associated issues such as frustration for doctors and the burden to the national health systems [6].

Analyzing all these problems we can classify them mainly into patient measurement monitoring and patient participation (understood as their implication and their relationship with the rest of participants). These issues are the main problem addressed in this work, involving an inefficiency in the HBP patient monitoring process that therefore reduces of the treatment effectiveness and increases the associated costs.

We propose to face these problems associated to the clinical process of high blood pressure treatment contributing to the improvement of the patient’s empowerment capacity. This concept has been accepted as an approach to improve the chronic processes management [7]. The main idea of patient’s empowerment is that the patient takes control in the decision-making process that affects his health. Of course, this requires that patients develop personal skills and have access to information and resources related with their health [8]. It is in the latter aspect, health information and resources, where technology can make the biggest contribution. For example, in [9] we can find as a conclusion of the study that blood pressure monitoring can help patients to improve the involvement in their own care management. A more regular monitoring of health variables is turned into more detailed information and allows patients to have more data that may contribute to the decision-making process that affects his health.

There are research projects in which devices like blood pressure devices, smart wrist bands or any wearable device are connected to mobile apps in order to monitor parameters like blood pressure [10] or physical activity [11]. All of them aim to assist patients to self-manage their health and/or lifestyle behaviors but most of them lack integration with the clinical process and they work in a standalone mode. Most of these proposals follow a bottom-up software design strategy, focused on resolving a specific problem achieving technological partial solutions from client requirements. This approach is not process-oriented, so it does not consider the strategic goals, the participants involved or the relationships between different parts of the overall clinical process. By contrast, a top-down process-oriented approach, focused on an overview of what the organization is trying to achieve, defines or re-designs the whole clinical process identifying specific problems that impede optimal results [12]. In this way, it is possible to offer a global and comprehensive solution focused on the needs of all involved parties.

For decades, several strategies and paradigms have been developed in other areas such as manufacturing and business, for the continuous improvement of both processes oriented to client’s satisfaction and process automation. Business Process Management (BPM) is one of the most recent process management strategies and also one that has a significant impact [13]. It is focused on the continuous improvement of business processes using information technology as one of its main principles in processes automation; furthermore, BPM enables alignment between business process and underlying technologies, integration of every actor and the complete tracking of the process execution. So far, in health issues, most research has focused on improving administrative processes, although there are some projects focused on improving clinical processes [14,15]. All these features are achieved in the form of a continuous process management life cycle consisting of the following phases: (1) discovery, where the current process and modeling are understood (AS_IS process); (2) analysis, where weaknesses and shortcomings are identified to reach new objectives; (3) (re) design, where a new process model (TO_BE process) is proposed with solutions to achieve new objectives; (4) implementation, where from the TO_BE model, workflow and tasks are integrated with the underlying technologies and participants; (5) execution, where process instances are initiated and interact with the participants; and (6) control, where it is validated if the proposed objectives are being achieved.

Associated to the BPM paradigm, there have arisen software platforms denominated Business Process Management System (BPMS), which implement their whole cycle and offer a set of techniques and technologies that make those stages more dynamic. The technological components linked to this system grant its key features and benefits: to monitor and control key performance indicators of the process; to guide participants automatically about the tasks they must perform; to reduce costs by automating and improving processes in real time; to adapt processes to changes in an agile way; to integrate process tasks with any technology; and to offer a solution decoupled from the underlying technologies.

The proposal and novelty of this paper focuses on a new model whose goal is to help in the improvement of the patient’s empowerment; to achieve this goal we have followed a top-down approach using BPM as backbone to take advantage of its benefits. With this philosophy, patients can know how context, environment and their physiological state influence their disease, and this knowledge will improve patients’ capability to become an active part in the decision-making process together with the health staff. All participants involved in the process can know through of standard mode what task to do, and when and how to do it. In addition, the HBP patient monitoring process will be adapted automatically from the information obtained and from the health staff actions. For that, in our proposal, we include a comprehensive redesign of the clinical process of hypertension integrating remote sensing, Internet of Things (IoT) solutions and usability principles.

Although we can find proposals which tackle, in a specific and independent way, several issues related to the hypertension process, there are not complete solutions. On the one side, most of the proposals present systems aimed just at the monitoring of the blood pressure focused in the viewing of the information or its transmission, but they don’t use context information or other data related to the disease (such as physical activity or weight changes) in order to give structured knowledge to the users (patients and doctors). On the other hand, some works are focused in a process approach using evidence-based guidelines, however these proposals are directed at solving problems from the point of view of clinical staff, especially human errors. There are not proposals that join both positions and their benefits. Our proposal adopts a process approach (using BPM) and a system that monitors several variables related to the hypertension so we can provide the patient with detailed dynamic information, dynamic recommendations according to the data obtained and, in general, a mechanism to guide patients and clinical staff in the tracking of this chronic disease.

In the following section, we study the state of the art of topics related with the research. Then, in Section 3 an analysis of the clinical process associated with hypertension and a group of detected weaknesses are presented. In Section 4 we propose a model focused on the improvement of patient’s empowerment capacity in order to minimize the detected weaknesses and, therefore, to contribute in the improvement of the quality of life of the patient. In Section 5 we present a redesign of the clinical process studied, integrating the proposed model. Next, an architecture aimed to help to the patient’s empowerment capacity is developed, using remote sensing and IoT to get health data (such as blood pressure) from patients. Using this architecture, in Section 7 a prototype has been designed and implemented. Finally, in Section 8, the conclusions of this paper are presented.

## 2. State of the Art

Nowadays, there are many factors to consider in order to monitor hypertension [16]. The remote measurement of blood pressure through specific devices connected to a smart device is a current topic that benefits most patients. Some studies have reported improved blood pressure using mobile health (mHealth) solutions [17]. In [18] a phone-based reminder application for patients who are using at least two prescription medications is used. This application works standalone without any integration with the clinical process. In [19], they tele-monitored blood pressure using a mobile device, and both patients and physicians were informed about data, but the system was focused just on alerts and not in the integration with the clinical process. In addition, this system does not inform about how to neutralize those alerts.

On the market, there are systems that collect and transmit patient’s information. Among all existing wireless technologies today, Bluetooth and RFID technologies are the most commonly used ones because they are user-friendly and not limited by patients’ devices [10]. In addition, these technologies are already available in many personal devices such as smartphones and tablets. Data transmission between devices and external information systems is usually achieved through a landline broadband or cellular network and security is ensured by encryption protocols (S-HTTP or S-FTP). Table 1 presents a summary of the most common consumer-level devices (including wireless communication) designed for self-monitoring of different health variables such as blood pressure, physical activity and weight.

If we talk about a clinical management process, current models focus on process-oriented IT technologies which have failed in meeting the requirements to integrate process support, information management and knowledge management. Current workflow management systems offer a promising approach for implementing site-specific organizational processes, but there are still lots of features missing [20]. They still do not have the ability to provide the flexibility, agility and continuous improvement required to optimize clinical processes. However, Business Process Management (BPM) provides methods, concepts and tools to fill these gaps [21].

In addition, IoT is a powerful technology that can improve all these features in healthcare and medicine. IoT has been applied to interconnect available medical resources and provide reliable, effective and smart healthcare services to patients with chronic diseases. IoT could enable patients that are not ill enough to be admitted to hospitals, prevent and get some information about early detection of signs of deteriorating health, and could allow them to get earlier and more efficient responses and treatments [22]. Furthermore, data collected by sensors can provide valuable information from the monitoring of those chronic diseases and that information may help some patients to make decisions about their healthcare.

IoT is providing innovations for the use of basic nursing care, but they are emerging and are still in early stages. This technology is yet vaguely adopted in nursing and their possibilities are not yet exploited as well as they could [23]. For this reason, nursing science might benefit from deeper involvement in engineering research. In [24] critical factors are proposed to determine potential users’ value perceptions and acceptance of IoT healthcare services. In [25], data is collected through body medical sensors to study medical monitoring with an application based on IoT. In [26] a system for chronic patient monitoring based on IoT, also capable of workout routine recommendations, was developed. The main limitation of the mentioned works is that they are presented as isolated solutions that are not fully integrated in the current clinical processes. Many solutions use proprietary software that does not allow integration with other tools or workflows. Therefore, these solutions would need some vertebral element so that they could be included completely in the clinical processes.

Environmental variables, such as pressure and temperature, can directly affect hypertensive patients [27]. Therefore, this work not only considers the patient’s monitoring through digital devices, but also the monitoring of the environment through different sensors. This automation of patient and environmental monitoring, acquisition, and management of data, might lead to improve the quality of healthcare by empowering the patients throughout the entire clinical process.

## 3. Hypertension Clinical Process

The main objective of this work is to propose a new model whose goal is to help in the improvement of the patient’s empowerment, in order to reduce the problems associated with the monitoring of patient measures and the participation of patients in the clinical process. According with a BPM strategy (in the discovery stage) we need to analyze how the disease is currently managed (known as the AS_IS process), including tasks performed, people involved, and associated roles. The objective of this stage is to detect where we should make the contributions in the current process in order to propose suitable solutions.

The presented process has been based on recommendations from doctors and different medical guidelines, worldwide accepted such as guidelines from the United Kingdom [16] or the United States [28]. From the study it is gathered that, although the guidelines define a group of common tasks, each of them establishes sets of subtasks or specific procedures according to the environment, where the implementation is developed (geographic location, primary care centers, hospitals, etc.), which shows a lack of a de facto standard. Besides, these guidelines are presented as procedures (oriented to the development of the task itself) much more than as processes, which are focused on patients and how to add value to satisfy their needs.

The current hypertension process involves several human teams participating in the different tasks, which implies the need of adequate coordination among them in order to avoid problems associated to patients. Apart from the patient, who should follow the clinical recommendations and to assist to appointments, we find two other roles: doctors and nurses.

**Figure 1:**
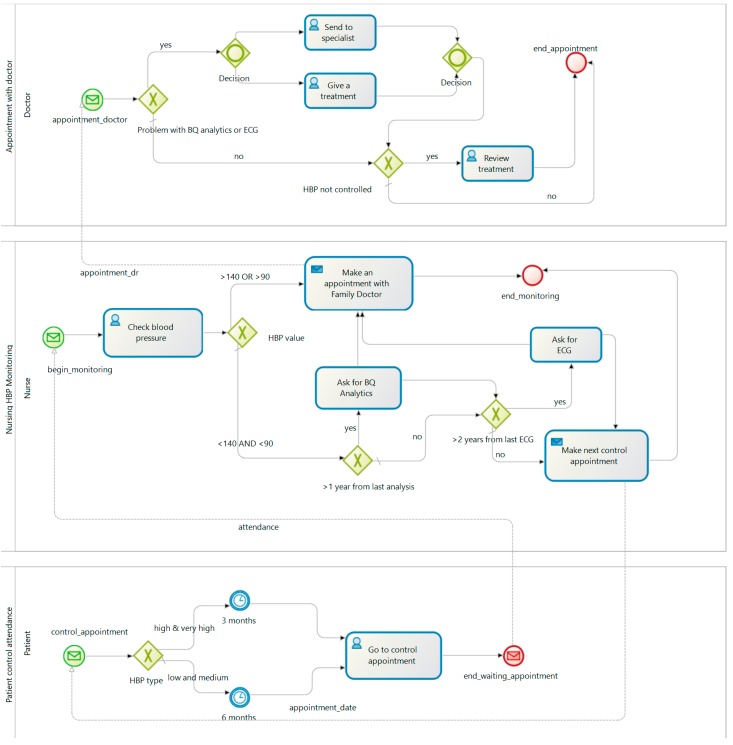
AS_IS process of HBP diagnosis and monitoring.

According to [16,28], when a person is diagnosed with high blood pressure (HBP), the treatment and monitoring process start in order to control its blood pressure. This patient must be classified according to their symptoms. This involves some tests and physical explorations. The nurse then takes blood pressure and depending on the severity of the illness, an analysis and an ECG may also be performed. According to the severity of the HBP, the patient initiates a drug treatment which is regularly supervised. All this treatment and monitoring process usually requires the patient goes to a health center. Some patients can provide the data collected when their blood pressure is taken at home. Similarly, the nurse measures the blood pressure to the patient when he is in the health center. Figure 1 shows the HBP clinical process AS_IS.

**Figure 2:**
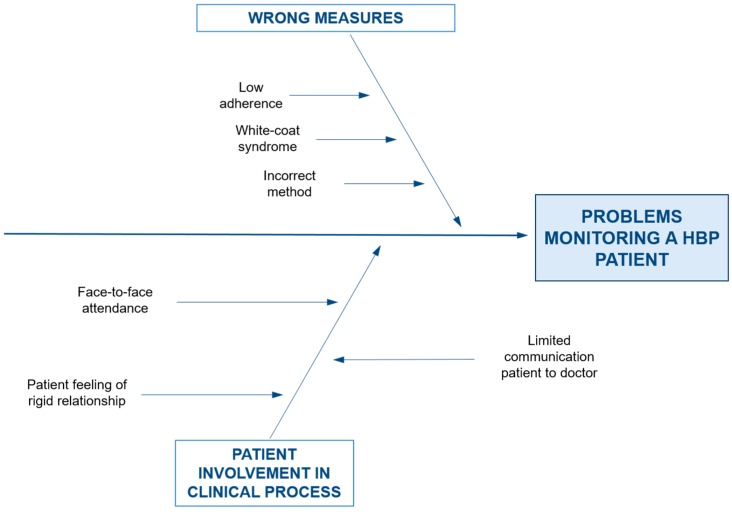
Ishikawa diagram for weaknesses identified in the HBP process.

Once the AS_IS process is analyzed, we identify the main causes that we want to minimize to achieve a more efficient HBP patient monitoring process. To identify the causes related to this problem we are going to divide them in two main groups: those directly associated to the measuring action and those related with the patient involvement in the clinical process. In Figure 2 we can see graphically in an Ishikawa or fish-bone diagram the identified weaknesses.

In the group of activities related to measurement actions we find low-adherence, white-coat syndrome and an incorrect measurement method. The first one—low-adherence—is a common problem in many medical treatments that imply a continuity in time; patients are busy or tired and they don’t find a good moment to go to a doctor’s appointment or the nurse office to measure the blood pressure. The white-coat syndrome is a well-known problem associated to blood pressure measurements: blood pressure increases because the measure is taken in a clinical environment by a person with a white coat (a doctor or a nurse). Finally, values taken from blood pressure measurements can be misinterpreted. When the nurse takes the blood pressure of the patient at the health center, the patient may be influenced by other factors such as family problems, labor problems, stress and many other factors. An evolution of blood pressure over time would be much more reliable so that the medical team could have a more accurate view of the evolution of hypertension in the patient.

In the second group of weaknesses we can find those directly related with the current model of monitoring and diagnosis, sometimes with a limited involvement of the patient in the clinical process. In many national health systems, due to budget constraints, a high ratio of patients per physician limits the time of a consultation; this lack of time can create in the patient a wrong feeling of disinterest by the doctor or a rigid relationship (due to the lack of time to explain clinical decisions to patients). Furthermore, the face-to-face attendance to doctor and nurse’s appointments increases indirect costs and limits daily monitoring (patients cannot go every day to the health center). Although doctors have the clinical knowledge to treat the disease, information and comments from a patient can be useful to adapt a treatment and to improve the personalization of the treatments; we should bear in mind that usually the person who best can know a patient is the own patient. Doctors doesn’t have the necessary resources and time to do a continuous monitoring of a patient and to adapt a completely personalized treatment for each patient. In order to solve these problems a new model is proposed in the next section.

## 4. Proposed Model

After analyzing the current process of hypertension monitoring and identifying the problems and weaknesses associated with the clinical process, in this research we consider as a starting point the achievement of the following objectives:

- To increase the flexibility of the doctor-patient communication processes, minimizing face-to-face appointments and enhancing telematic interaction.
- To incorporate into the clinical process all the information associated with the disease to perform a correct monitoring and to optimize the treatment.
  ○ Get the physiological variables in patient’s environment.
  ○ Increase the frequency of information monitoring, to have the most up-to-date information possible at each moment.
  ○ Get information in the most simple, transparent, automatic and unattended manner.
  ○ Make this information accessible at any time and from anywhere to all participants.
- To reduce costs associated with the monitoring process.
- To improve the patient’s quality of life.
- To improve adherence to treatment.
- To encourage and involve the patient in the monitoring of his disease and in decision-making.

Our proposal consists of a new model whose main goal is to help in the process of empowering the patient. Through empowerment we get the patient to have all the information regarding the evolution and treatment of their disease. With this philosophy, the patient can know at every moment the different possibilities he must face during the treatment, he can know how his context and environment may influence his illness, and he may become an active part in the process, participating in the decision-making together with the health staff.

In our proposal, the help to improve the empowerment capacity is carried out through three fundamental concepts: objectives, variables and recommendations.

The *objectives* consist of goals or stages that the patient must achieve to improve in the evolution of his disease and are proposed by the medical staff. Examples of goals are: to reduce weight, to follow a proper diet, to avoid stimulating substances or to stop smoking. With the information that the doctor has about the patient, he can modify his objectives at any time. Patient’s empowerment capacity is improved with the possibility of the patient to decide in each moment what objectives he wants to achieve. This may promote treatment focused on the objectives agreed by the patient, may increase their involvement in the process, encouraging improvement actions and, therefore, may improve treatment adherence.

In order to achieve the negotiated objectives, and as one of the main contributions of the proposal, the monitoring of both, the patient’s physical and environmental or contextual *variables*, the analysis as value-added information and the ubiquitous and real-time provision to all participants is fundamental for the model. This information allows professionals to propose more appropriate objectives to patients, and patients to take suitable actions to the accepted goals.

To assist the patient in their empowerment process, an automated system that, based on accepted objectives and monitored variables, offers the patient a set of *recommendations* is proposed. The recommendations consist of advice, guidelines or actions that the patient must perform. The recommendations are dynamic and they change when the objectives or variables monitored change.

The proposed model is distributed and is composed by the medical team, the patient, the environmental sensing system and the empowerment system, elements that are highly decoupled in the model. In addition, it clearly identifies four flows of information that flow between the different elements and operate independently, providing a high level of scalability in the model and great autonomy to all parts involved in the system.

**Figure 3:**
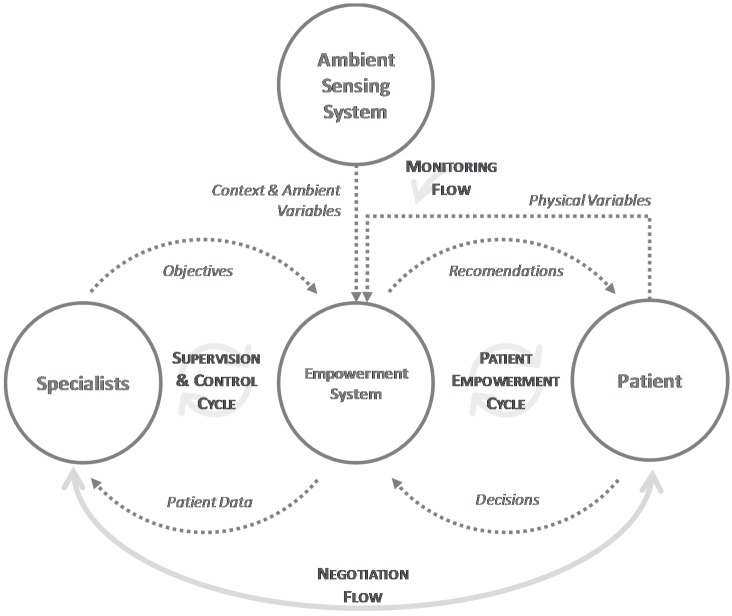
Management model of hypertension based on patient empowerment.

Figure 3 shows the proposed model, including all the elements that compose it, the exchanges of information they perform and the main flows. These flows represent functional processes in the model, where information is processed, communicated, and interpreted by the different components of the model. Next, the flows are described.

Through the *negotiation flow* the patient goes from being a passive element in the process to an active element. The negotiation is a bidirectional flow carried out by the objectives, which are proposed by the medical team and accepted or refused by the patient. Negotiation is the starting stage for the model and it will determine the rest of the process.

The *monitoring flow* allows the system to obtain all variables related to the clinical process of the disease. Information is obtained from the patient, such as weight, blood pressure or mood, or it can be environmental, such as temperature or atmospheric pressure in the context of the patient. The variables can be taken automatically, such as the environmental ones from a remote monitoring system, in an assisted manner, such as the physical exercise performed by connecting to a smart band using Bluetooth, or in a manual manner, such as forms to introduce the patient’s mood.

The *patient empowerment cycle* completes patient integration into the system, assisting the patient to achieve their goals. For this, the empowerment system, based on the decisions made by the patient and the monitored variables, generates a set of recommendations that will guide the patient in his actions. For example, if the patient has agreed to lose weight, the system will provide recommendations on exercise and diet. If the patient follows these recommendations, and their weight is reduced, when the empowerment system captures this change in the weight variable, it can provide other recommendations, to adjust the treatment to the new conditions. This provides a high flexibility and adaptability to the proposed model.

Finally, the *supervision and control cycle* allow the medical staff to obtain all the patient’s data, including the agreed objectives and the evolution of the associated variables. With this, the professionals can do a better monitoring and can establish a new set of objectives, that again will have to be negotiated with the patient.

## 5. Process Redesign

To achieve the model mentioned above, the empowerment model for hypertensive patients is based on the synergy of a set of paradigms, principles, techniques and ICT technologies such as BPM, Service Oriented Architecture (SOA), remote sensing systems, IoT solutions and principles of usability and user’s experience.

This proposal uses BPM as backbone of the model by integrating solutions whose union increases the benefits that each individually contribute. BPM is a strategy for the business processes management oriented to continuous improvement by using information technologies, whose benefits are fully aligned with the following objectives:

- First, it focuses on a top-down approach that allows one to design an IT solution based on the strategic objectives of the organization rather than the technological requirements, thus allowing the alignment between the patient’s needs and the appropriate treatment. In the proposal, it has allowed the design of a new HBP clinical process based on the current guides but aligned with the objectives and needs defined in the proposed model, regardless of the technology.
- This modelling is done through a standard notation of process modelling called Business Process Model Notation (BPMN) that is intuitive and understandable for all those involved in the process. Thus, BPMN is targeted to non-technical staff with the aim of being used by business experts in the field of application. This notation allows the normalization and the standardization of the process by avoiding ambiguities and discrepancies in how the HBP treatment process should be developed and who should perform each of the tasks. As a result, it increases the visibility of the process and the available information about its operation. For example, one of the sub processes performed by the patients is to check blood pressure. This sub process involves a set of steps to obtain a right blood pressure measure. Those steps are shown to the patient in a simple task sequence such as: *to remove excess clothing that might interfere*, *sit upright*, *put your upper arm level with your heart, to put feet flat on the floor, to take five minutes to relax, to position the stethoscope* and *to measure blood pressure.* From this simple sub--process modeled in BPMN, a BPMS can manage the track and trace of each step, validating if the previous steps are done before passing on to the next task. This could help to reduce wrong measurements due to the lack of expertise of patients, or avoid that health staff skip a step due to lack of time. However, unlike other proposals not based on BPM, this workflow logic would be outside of the application code.
- Another advantage added to BPM, specifically to the BPMN modelling, is the process composing capability. This feature allows flexibility in solutions to patients in a personalized way depending on their context. For example, following the previous case, a patient recommendation can have associated a single step or a sub-process. This sub-process could be *to obtain blood pressure* or *practice exercise.* Any of these sub-processes involve a workflow. Based on the recommendation that the patient must perform one or another sub-process will be discarded. Moreover, any new changes made by the physicians (in this case centered on clinical guidelines) would be easily incorporated by them through the modeling interface offered by BPMS. In this way, the HBP process will be adapted for each patient. In addition, when new recommendations will appear in the future, new sub-processes based in clinical evidences could be added from the BPMN model.
- BPM has an associated IT solution called BPMS that sustains its entire life cycle. BPMS is based on a set of decision tables easily parameterizable through the included modeling tools. One of the features of a BPM system is the possibility of automation of process task management from the BPMN model. In this way, all the participants involved in the process will be assisted in the execution of the treatment, knowing always the tasks that must be performed and the state of the entire process.
- Another feature of BPM systems is the ability to integrate technology and human resources through connectors, paradigms such as service-oriented architecture (SOA) and a user interface design (human tasks). It is at this point that the use of other technologies, techniques and principles of design will allow to increase and complete the benefits of BPM through the incorporation of IoT solutions, remote sensing and usability aspects into service tasks and human tasks. The underlying technology could be proprietary or integrated from legacy or third parties’ systems. For example, the proposed model includes the *Agree appointment with patient*, necessary to perform the HBP process, but this sub-process could be executed over any appointment system by means of integration platforms included in BPMS such as connectors, Enterprise Service Bus (ESB) platforms or Web Service technologies. Other example would be related with the integration of IoT technologies or remote sensors such as activity wristbands or smart scales through a SOA paradigm. In our model, part of the workflow will be deployed over these devices, launching an event when reading data. This event starts a monitoring HBP instance of the patient, which is exposed as a Web Service. In contrast, bottom-up functional oriented solutions, tend to create isolation and not include integration solutions with other systems [29].

Through the capacities described above, a redesign of the hypertension treatment process has been carried out and it implements the proposed empowerment model aligned with the previously identified objectives, thus contributing in the improvement of the current process. To do this, we describe the redesign, the value that each of the decisions adds to the patient and the health system in general. Specifically, the solution is presented based on the initial diagnosis of the patient and the initial objectives to initiate the treatment.

First, two processes are introduced for the unattended acquisition of information on the patient’s physiological status and environment information. These processes have been called *Context and Ambient Variable Monitoring and Physical Variable Monitoring*.

**Figure 4:**
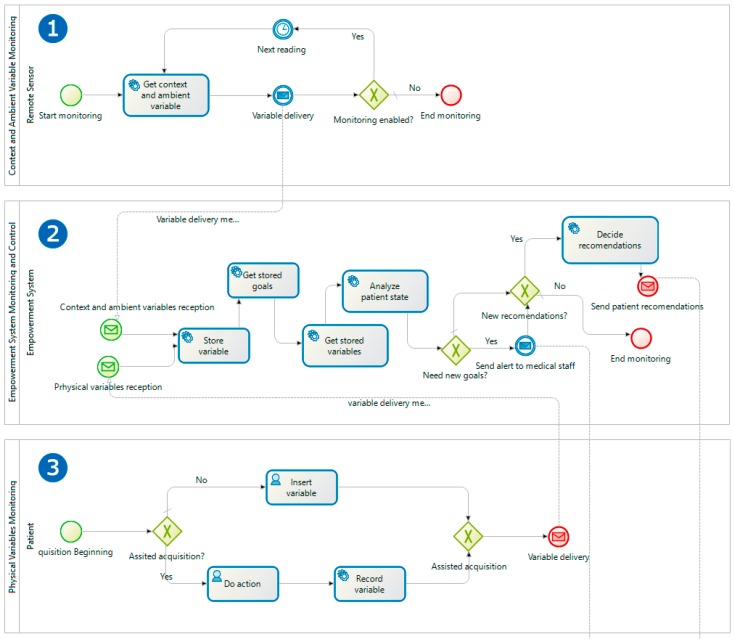
Process redesign (TO_BE). Patient empowerment cycle from variables monitoring cycle.

The first one (Figure 4(1)) includes a single task that is responsible for the periodic acquisition of the environment variables, such as temperature and pressure, while the service is enabled. This process will be launched for each variable to be acquired and for the environment of each patient, and it acquires the information through the integration with different remote sensing systems designed for this purpose that will be described later.

The second process (Figure 4(3)) allows the remote acquisition of the patient’s physiological information. Depending on the nature of the variables to be acquired, these can be of two types: assisted variables, where the patient will have daily devices to take their data with computational and communication variables, capable of sending the information acquired automatically, such as a digital scale, a smart band, a digital blood pressure monitor, etc.

Each time there is a new recording of variables, both environmental or physiological, an instance of *Empowerment System Monitoring and Control* (Figure 4(2)) is launched, which after registering the new variable, allows one to maintain a history of the state and environment of the patient, obtains the rest of variables and analyses them. Then, the system automatically decides if the patient has an anomaly or improvement that requires a face-to-face appointment with the medical team to modify the objectives, and if it remains the same treatment or if the performed changes need the sending of new recommendations. For example, in the latter case, it could be the situation where the outside temperature was high and the recommended physical activities had to be modified so far. The *Decide recommendations* task is based on a business rule engine that, depending on the variables and the objectives, establishes the recommendations to be followed by the patient.

**Figure 5:**
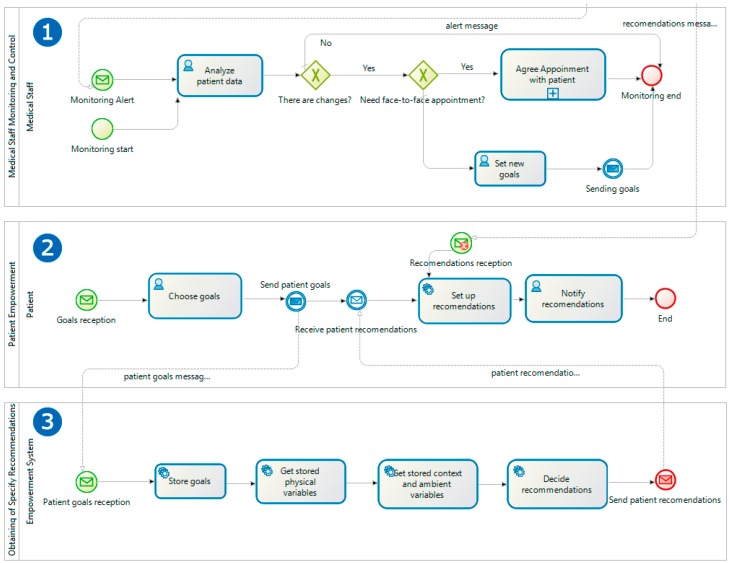
Process redesign (TO_BE). Patient empowerment cycle from medical staff control and monitoring cycle.

The rule engine is provided with a knowledge base previously given by health professionals of the Nurse Department at the University of Alicante. Once established, they are notified to the patient's system, in this case their mobile device, launching the *Patient Empowerment* process starting the task *Set up recommendations* (Figure 5(2)). This first redesign improves multiple aspects:

- Elimination of unnecessary visits to the health center and only set up a visit when the patient’s state requires it. This can eliminate unnecessary displacements, which may mean an improvement in the patient's quality of life. In addition, it may mean cost savings in the health system.
- Regular and remote monitoring throughout the day to day activities of the patient, and not only in the visits to the health center, which may allow for a more precise and personalized treatment.
- Regular incorporation of variables of the patient's environment that can affect his illness, such as pressure and temperature. So far, they are not considered in the day to day activity of the patient.
- Daily automatic update of recommendations, helping offer a much more precise and personalized treatment.
- Integration of the patient in the process through usable interfaces and everyday systems that suppose little intrusiveness in their daily life.

The other part of the redesigned process focuses on the doctor-patient relationship, which changes from a passive model to a patient-centered model and its needs. Firstly, the main process called *Medical Staff Monitoring and Control* is invoked in two possible ways, either through the medical team itself interested in knowing the state of a patient and its evolution, or, as we saw in the previous process, being invoked by the *Empowerment System* Monitoring and Control Process (Figure 5(1)), before changes that require the modification of objectives. In both cases, the physician can perform a modification of the objectives through a web user interface and if it is considered necessary, to request an appointment for a face-to-face visit.

Here we are two important aspects that improve the process. The physician assumes a proactive role in the visits of the patient, since he has information available always and from any place in the evolution of the patient. Patient control and supervision processes can be performed during part of the time saved by avoiding face-to-face visits. For example, this can be integrated into the professional’s workflow by scheduling virtual appointments where such control would be performed. On the other hand, the patient participates in the decision-making about his treatment once the doctor has determined the new objectives, through the *Patient Empowerment Process* (Figure 5(2)). This process is launched when the medical team establishes a new objective proposal, which must decide what it is committed to. Once the objectives agreed upon by both parties are sent to the empowerment system that launches the *Obtaining of Specific Recommendations* process (Figure 5(3)). The process, once it updates the new objectives, obtains the most updated physiological and environmental variables of the patient and performs the analysis to determine the recommendations to be made by the patient. Once established they are sent to the patient, who will be notified through his mobile device of the new conditions. This redesign offers:

- Regular remote monitoring of the patient so that the medical team can plan visits based on current information.
- More information available for the decision-making of health professionals, also counting on contextual information of the environment and all the information of their daily habits.
- Dynamic and agile adaptation of the objectives and associated recommendations depending on the patient’s condition.

## 6. Empowerment System Architecture

Given that the proposed model consists of a highly-distributed system, it is proposed, as a general framework for integration, the use of a SOA using Web Services based on Representational State Transfer (REST) architectural style, also called RESTful services. These services have a low level of coupling and a data-focused orientation, which makes it suitable for the proposed model.

**Figure 6:**
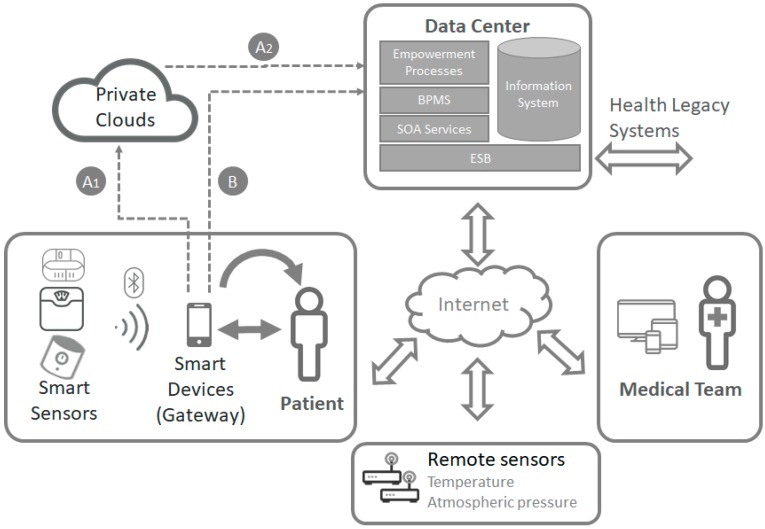
System Architecture.

In the proposed architecture (see Figure 6) there is a data center where the information system, the main services that give access to the information, and a BPMS to manage the process life cycle and the empowerment system, are located.

Another important issue is the semantic integration [30] that would allow the interchange of data with an electronic health record system. Since we have not connected our system with an electronic health record system, we have not implemented any standard; anyway, the architecture is ready, thanks to our approach is based on an ESB platform that increases the integration characteristics of the BPMS by means of standard connector (HL7), data and message transformer patterns among others [31].

**Figure 7:**
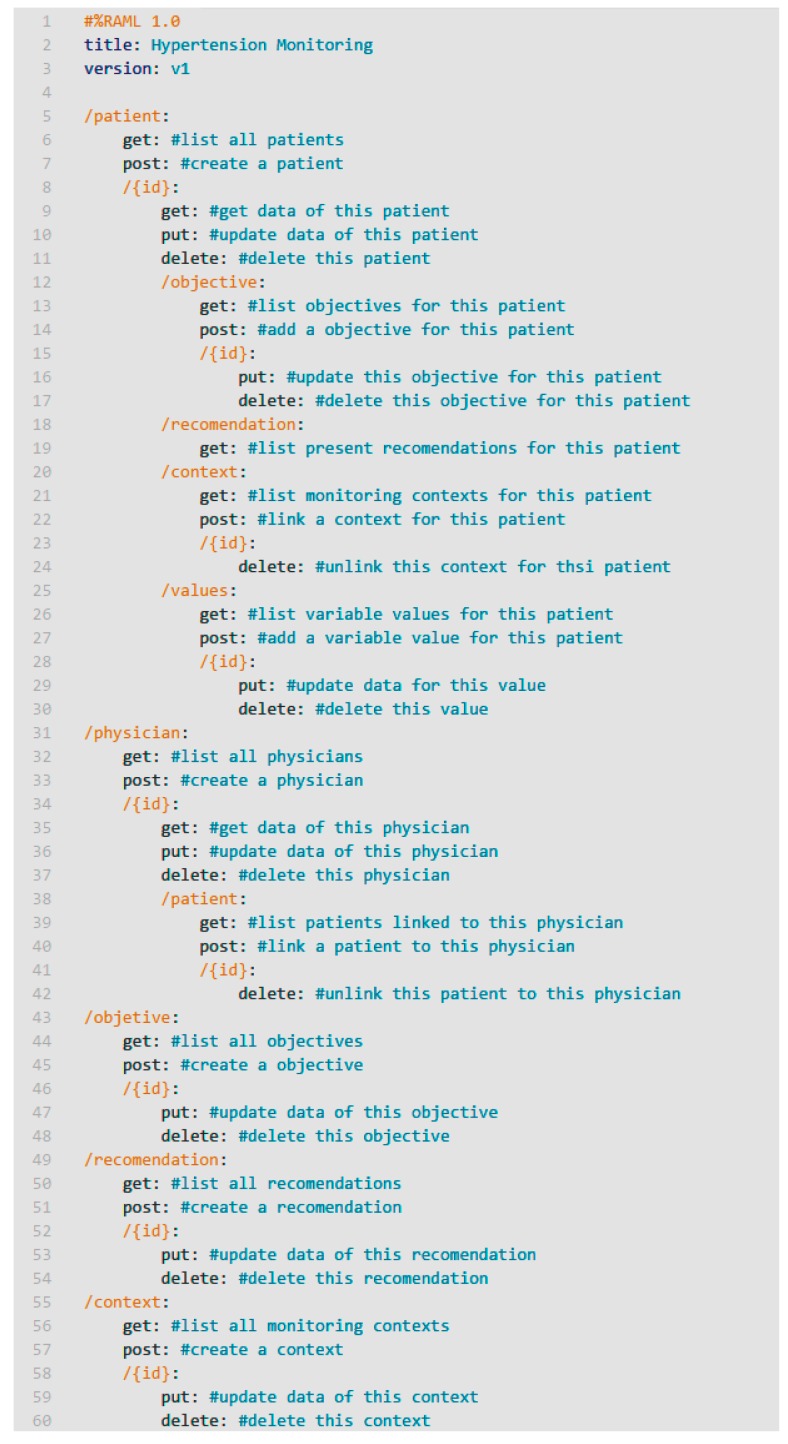
RESTful API definition in RAML.

As a starting point, we have defined the web services interface that will use all the components. To do this, we first identified the entities of the information model collected in our proposal: patients, professionals, objectives, recommendations, contexts and variables. Figure 7 shows a summary of the main endpoints and methods of the RESTful services.

Through these services, all the fundamental tasks of the model can be performed: associating physicians with patients, associating patients to monitoring contexts, proposing and accepting objectives, establishing recommendations, entering variable values and, of course, consulting any information associated with entities. For the monitoring process, when the variables of a patient are request, both those associated with the patient, as well as those of all the contexts to which the patient is related, will be obtained. This allows the system to have different monitoring contexts (for housing, neighborhood, city or area) covered by a remote sensor of environmental variables that will be shared by several patients. In addition, it is possible to incorporate external monitoring services into the system, such as public monitoring services.

To do the monitoring of patient variables, smart sensor devices like blood pressure devices, smart wristbands and scales have been integrated into the system. These devices have their own operating interface, easy to use by patients. Once the data is collected, it is sent to a smart device like a smartphone or tablet through Bluetooth or Wi-Fi communication. To access to this information there are two options, depending on whether the smart sensor is a private or an open system.

In the case of private systems, information is usually sent by an app to a private cloud (A1 path in Figure 6). Then, to obtain this information, the monitoring service in the data center will have to request this data to the private cloud (A2 path in Figure 6).

On the other hand, if patients use open systems, the smart device would be able to access this data directly and then to send it to data center (B path in Figure 6).

In the case of variables that cannot be monitored in an assisted way, a set of wizards and forms are incorporated in the smart device that help the user, through highly usable and accessible interfaces, to enter that information. These variables, like the rest, are sent to the information system through the monitoring services to be integrated into the empowerment process.

To this end, and since the clinical process has been modeled using BPMN, a BPMS has been used to support the life cycle of the clinical process. This brings a high flexibility to the system, since changes in BPMN are automatically reflected in the process. Thus, BPMS is the core of the system, coordinating all the tasks that compose it, as well as the flow of information that make possible the empowerment of the patient.

Finally, the medical staff will have a management system, with which they can monitor the patient's clinical process at any times, obtaining the evolution of their variables, their selected objectives and their preferences. In addition, at any time, the doctor can modify the goals proposed to the patient, to start a new negotiation process.

## 7. Testing and Validation

In order to validate the proposal, a prototype that cover all the components proposed in the model and architecture has been implemented. Subsequently, and based on this prototype, a series of experiments have been carried out that validate the adequacy of the proposal.

### 7.1. Information System and Services

For the implementation of the information system a *MySQL* database has been used, where the data model identified in previous sections has been designed.

For the development of the services *NodeJS*, an execution engine for JavaScript, has been used, along with *Express*, a framework for web services. To allow access from services to the database the *mysql* module for *NodeJS* has been used.

Services implement the API defined in Figure 7, and they are transported in a secure channel (*HTTPS*) in order to provide security and privacy.

### 7.2. BPMS

To support the life cycle of the proposed BPM model, Bonita Community Edition version 7.4 software has been used. Bonita is an open source and modular BPMS that implements the full BPM lifecycle. With this tool, and in collaboration with a medical team, all procedures associated with the clinical process resulting from the proposed redesign have been designed. The execution of these processes allows the empowerment system to offer appropriate recommendations to each patient, depending on their objectives and their associated variables monitored.

### 7.3. Patient Monitoring System

**Figure 8:**
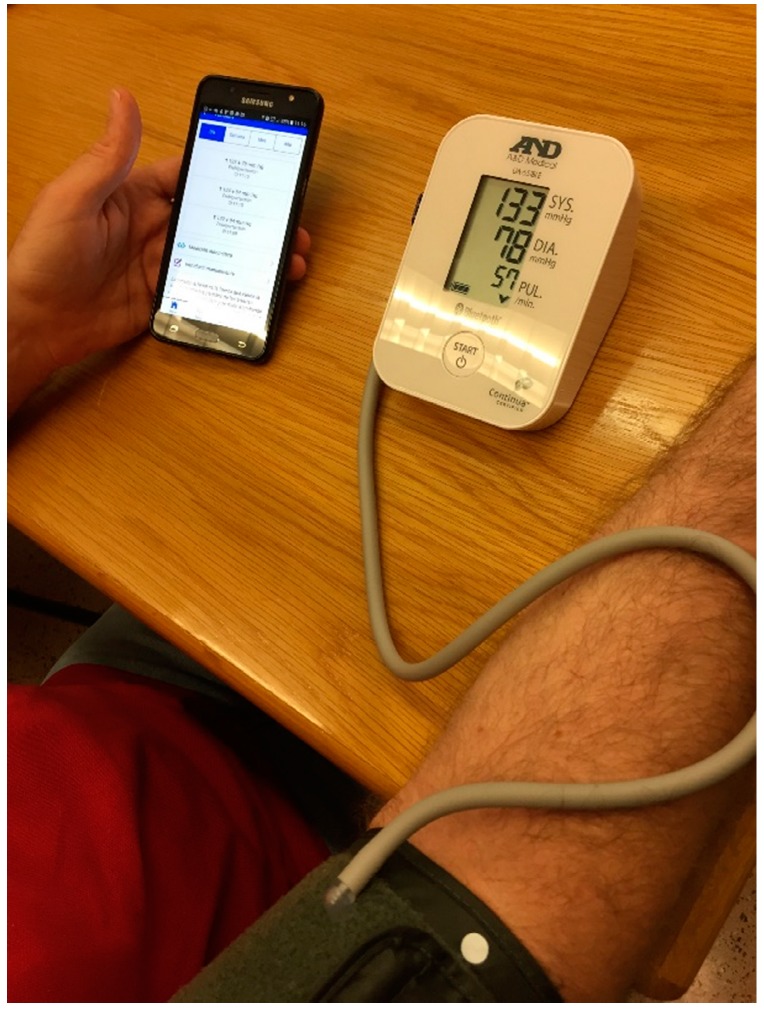
Patient monitoring system sensing blood pressure.

**Figure 9:**
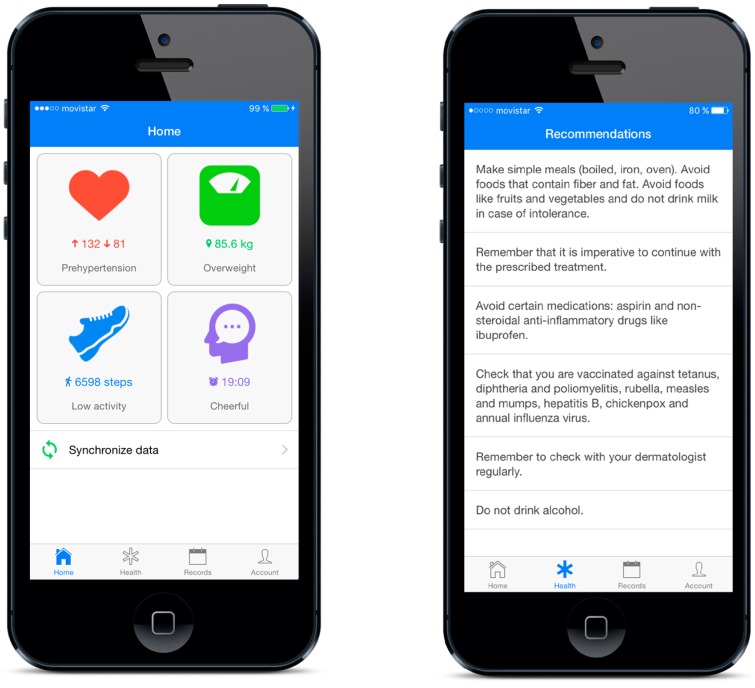
Mobile application views: main view (**Left**) and recommendations view (**Right**).

The application is cross-platform and has been developed using the mobile application development framework Apache Cordova. During the tests, the patient will have a smartphone as a data acquisition device, whose function will be to intermediate between the medical devices and the core system. As mentioned above, communication will be via Bluetooth LE and by manual entry of data by the patient. In this work, we use a model UC-651BLE digital blood pressure and a model UC-352BLE digital scale, both from the A&D Company (Tokyo, Japan). Figure 8 shows an example of blood pressure test with our Android application and the mentioned digital blood pressure monitor. The smart band used is the Fitbit Flex model, which works with its own web API. The mobile application will record all this data and will provide information in the form of personalized recommendations. These recommendations are framed in different healthy habits related with the patient preferences such as physical activity, diet or restrictions given by the disease. In addition, the application will notify the patient in case of detecting any risk factor. Figure 9 shows the main view of the mobile application where patients can monitor every variable, and the recommendations view where patients can visualize the current recommendations based on their preferences, objectives and monitored variables.

### 7.4. Context Monitoring System

In order to capture the environmental information, we have developed a prototype of a monitoring system for temperature and atmospheric pressure, two variables that affect hypertensive patients.

For this, a Raspberry PI v3 unit has been used as hardware platform, since it is robust and has a low power consumption, which makes it suitable to support a service that works continuously, is flexible, allowing the connection of various sensors, and has wired and wireless communication interfaces, necessary to transmit monitored information.

**Figure 10:**
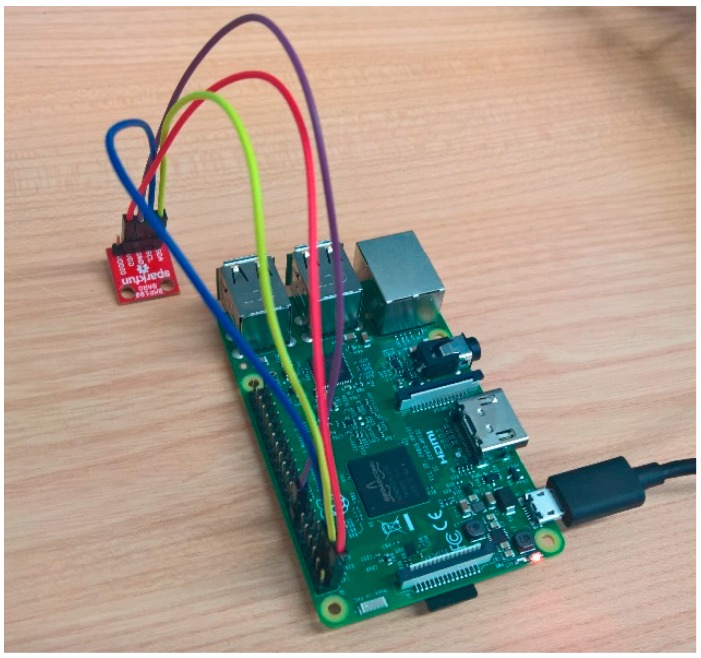
Prototype of the context monitoring system.

A BPM180 barometric sensor model, a low-power module that measures atmospheric pressure in a range between 300 and 1100 hPa with a margin of error of only 0.02 hPa has been used. This module also has a temperature sensor. The sensor uses an I2C interface, and can be easily connected to the Raspberry Pi. In Figure 10, one can see the implemented prototype.

**Figure 11:**
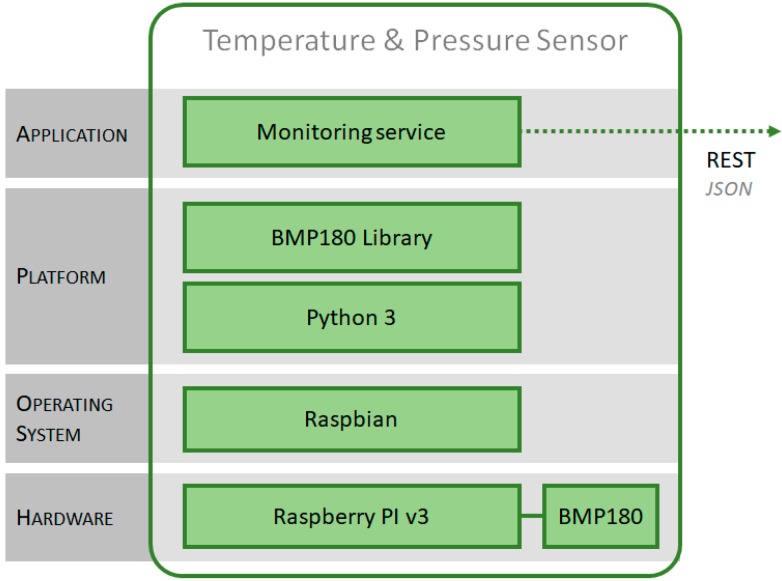
Architecture of context monitoring system.

For the software layers (see Figure 11), Raspbian has been chosen as the operating system, and Python as a development platform, where the monitoring service has been implemented.

This service obtains the variables from the sensor through an access library and transmits them to the data center. For sending the information, the sensor uses a PUSH model, consuming, as a client, the web services implemented in the data center. The monitoring system allows to configure all the parameters of the service: sampling frequency, address of the service, identifiers of context, etc.

**Figure 12:**
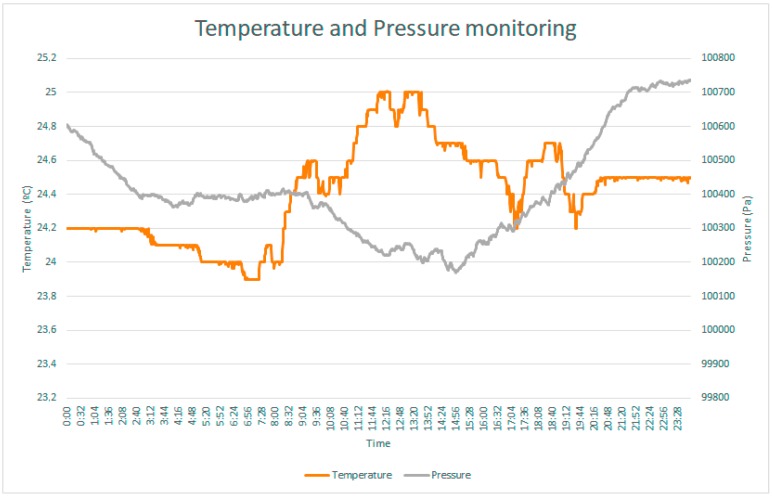
Evolution of temperature and pressure monitored by sensor prototype.

As an example, in Figure 12 one can see the values of the temperature and pressure variables monitored for 24 h.

### 7.5. Medical Web App

**Figure 13:**
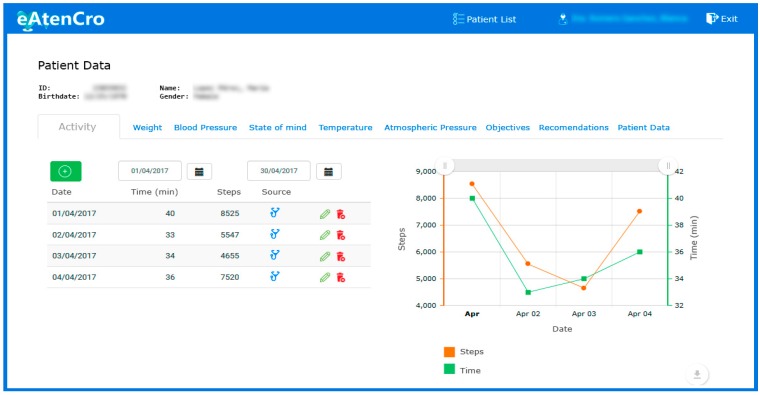
Screenshot of the web application for medical staff.

For management and supervision by the medical team, a prototype web application has been developed (see Figure 13). The application is made according to the current web standards: HTML5, CSS3 and JavaScript ES6. For the web development, we have been used the AngularJS framework, along with Bootstrap for a responsive interface that facilitates its access from any device connected to the Internet. This web app, as backend, consumes the services offered by the system, and it has been developed following usability patterns and accessibility guides that facilitate its use by users.

### 7.6. Experiments

Once the prototype was developed, 12 patients were selected to perform a set of experiments using the proposed system. The experiments were mainly directed at knowing the opinion of the patients about the degree of empowerment that the system helped the patients achieve. Assisted by health staff a set of goals to be achieved by patients was defined, a set of recommendations was established and which recommendations are provided for certain objectives and data monitoring was defined in the BPMN. The experiment lasted two months. Participants were previously given monitoring devices and received instructions on their use.

After the experiment was carried out, it was verified that in both cases, when the patient changes his objectives and when changes occur in the monitored variables, the system modifies the recommendations for each patient. The health staff analyzed these recommendations updates and validated them as correct. This test validates the flexibility and generality of the proposal.

**Figure 14:**
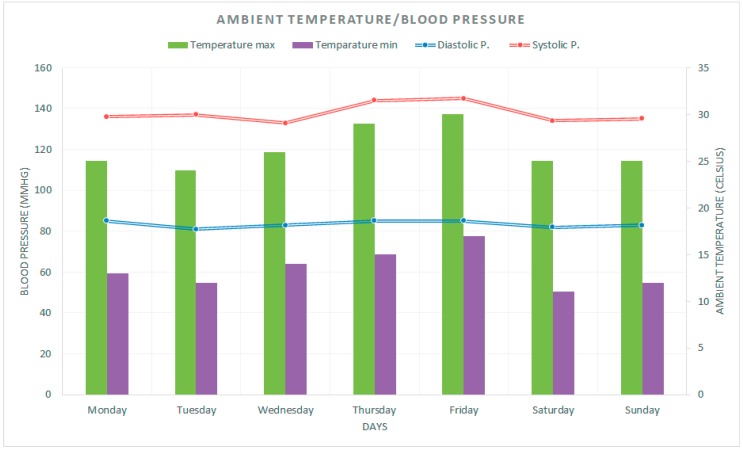
Blood pressure and ambient temperature measurements.

Next, to illustrate the experiment, we explain an example using the developed prototype. In Figure 14 we can observe the evolution during a week of the blood pressure (diastolic and systolic) and the ambient temperature. With that simple information, the system is very useful for the patient in different ways (bearing in mind the empowerment of the patient). For example, it can help the patient in a later analysis of the blood pressure because on Thursday and Friday, blood pressure has been higher than other days (and higher than it is appropriate) but the higher blood pressure coincided with a higher temperature so, maybe, the patient has done something that together with the temperature negatively affected the blood pressure. Furthermore, with the real time information on ambient temperature, the system will recommend avoiding vigorous exercise such as going running.

In addition, a short test was performed asking the participants about aspects related to the experiments. For each question, the participant answered with a numerical value between 1 (strongly disagree) and 5 (strongly agree). Next, the items of the test and the average value of the answers are presented.

- The app is easy to use: 3.67.
- The process of variables monitoring is easy: 3.33.
- I feel better informed about the evolution of my disease: 4.67.
- I have more control over my disease: 4.42.
- Overall, I am satisfied with the system: 4.58.

The results show the appropriateness of the proposal, and how it may help to improve the patient’s empowerment capacity, in the sense that he/she feels better informed and with more control over their disease.

Patients are constantly informed by the system about their conditions, not only with their own data but with data provided by the environmental devices. Thus, by providing this structured information, patients can make properly informed decisions. Moreover, these decisions are also based on their goals and the system automatically adapts the recommendations according to their preferences to achieve each goal in an easy and non-invasive way. The system also informs the medical staff of the evolution of the disease in a continuous way, thus making the patients safer because they can perceive that the disease is permanently under their control.

## 8. Conclusions

The inefficiency problems associated to clinical processes of chronic diseases, and the lack of integration among medical staff, patients and the process itself, are goals to be achieved through the proposal of a new model based on the BPM strategy, IoT, remote sensing and usability principles. BPM is shown as the keystone of the proposal improving its benefits through the synergy with other technological solutions.

As a result of the model, a redesign of the clinical process associated to the hypertension has been obtained, which presents the following novelties and characteristics:

- The proposed model is based in a top-down approach that enables achieving a massive treatment range in an unattended way.
- The proposal offers a flexible model which, thanks to the availability of the information about the health of the patient and the context in real time, may help doctors provide accurate treatment in every moment.
- The inclusion of information about the ambient and the context of the patient, that is not considered during the treatment of the patient at present.
- To reduce unnecessary appointments at the health center, which may improve the quality of life of the patient and can reduce costs to the national health service.
- To fully integrate the patient in the clinical process, helping him to participate in the decisions about his/her illness.
- To provide a model that guides the patient along the clinical process, helping him/her to improve his/her empowerment capacity.

Finally, we have developed an architecture to include the proposed model into the clinical process, adding health sensors to monitor physical variables and to obtain data related to the style of life, and remote sensors to obtain useful information from outside patients, such as ambient temperature. All this information help patients to take their own decisions in relation to the disease. The main benefits of the proposed architecture are the use of an SOA approach that provides integration with low coupling, scalability, reusability and interoperability.

To test the architecture, a prototype has been implemented using a scale, a pressure monitor, a smart band, an ambient temperature and a pressure sensor. We have also developed the corresponding mobile and web application to gather the data and analyze the obtained information. Future work will address mechanisms to improve the negotiation process, guiding and directing the patient appropriately in decision making.

## Figures and Tables

**Table 1:** Devices used to control hypertension parameters.

Brand	Model	Communication	App	Price	Modality	Device
Withings	Wireless Blood Pressure Monitor	Bluetooth	- Mobile - Web - Cloud	130 €	closed system	Blood Pressure
A&D Medicals	UA-767PBT-Ci	Bluetooth	- Mobile - PC - Web Cloud	157 €	closed system	Blood Pressure
Angel Sensor	m1	Bluetooth RFID/NFC	Mobile	99 $	open system	Activity wristband
Xiaomi	My Band	Bluetooth	Mobile	27.99 $	closed system	Activity wristband
Medisana	BS 440	Bluetooth	- Mobile - Web Cloud	89 €	closed system	Scales
A&D Medicals	AD-6121A	Bluetooth	- Mobile - PC - Web Cloud	520 €	closed system	Scales
